# Prediction of relapse after lymph node dissection for germ cell tumours: can salvage chemotherapy be avoided?

**DOI:** 10.1054/bjoc.2000.1613

**Published:** 2001-02

**Authors:** D M Berney, J Shamash, W F Hendry, A Arora, S Jordan, R T Oliver

**Affiliations:** Dept of Histopathology and Morbid Anantomy, Barts and The London NHS Trust, St Bartholomews Hospital, London; Dept of Medical Oncology, Barts and The London NHS Trust, St Bartholomews Hospital, London; Dept of Urology, Barts and The London NHS Trust, St Bartholomews Hospital, London

**Keywords:** embryonal carcinoma, Ki-67, lymph node

## Abstract

Salvage chemotherapy has been used by some oncology centres for patients with residual malignant or immature elements in retroperitoneal lymph node dissections removed for metastatic non-seminomatous germ cell tumours. However, surveillance of these patients shows that many are cured by surgery alone. 118 retroperitoneal lymph node dissections for metastatic non-seminomatous germ cell tumours were reviewed and the morphology seen within them was quantified. 28 of these had immature or malignant elements and had been treated by surveillance before administration of further chemotherapy. The proliferation rate in these cases was assessed by immunochemistry. The proliferation index and the amount of embryonal carcinoma (EC) were both predictors of recurrence and therefore the need for further chemotherapy. Patients with greater than 25% of EC had an 84% chance of relapse and those with a Ki-67 index of greater than 50% had a 71% chance of relapse. The two tests had a positive predictive value of 83% and 71%, respectively. Patients with such a high risk of recurrence could be considered for post-operative adjuvant therapy at this point whilst others would be suitable for a watchful waiting approach. © 2001 Cancer Research Campaign http://www.bjcancer.com


British Journal of Cancer (2001) **84**(3), 340–343
© 2001 Cancer Research Campaign
doi: 10.1054/ bjoc.2000.1613, available online at http://www.idealibrary.com on IDEAL®

### Prediction of relapse after lymph node dissection for germ cell tumours: can salvage chemotherapy be avoided?

**DM Berney[1], J Shamash[2], WF Hendry[3], A Arora[1], S Jordan[1] and RT Oliver[2]**

Dept of Histopathology and Morbid Anantomy[1], Dept of Medical Oncology[2], Dept of Urology[3], Barts and The London NHS Trust, St Bartholomews Hospital, London

**Summary** Salvage chemotherapy has been used by some oncology centres for patients with residual malignant or immature elements in retroperitoneal lymph node dissections removed for metastatic non-seminomatous germ cell tumours. However, surveillance of these patients shows that many are cured by surgery alone. 118 retroperitoneal lymph node dissections for metastatic non-seminomatous germ cell tumours were reviewed and the morphology seen within them was quantified. 28 of these had immature or malignant elements and had been treated by surveillance before administration of further chemotherapy. The proliferation rate in these cases was assessed by immunochemistry. The proliferation index and the amount of embryonal carcinoma (EC) were both predictors of recurrence and therefore the need for further chemotherapy. Patients with greater than 25% of EC had an 84% chance of relapse and those with a Ki-67 index of greater than 50% had a 71% chance of relapse. The two tests had a positive predictive value of 83% and 71%, respectively. Patients with such a high risk of recurrence could be considered for post-operative adjuvant therapy at this point whilst others would be suitable for a watchful waiting approach. © 2001 Cancer Research Campaign http://www.bjcancer.com

**Keywords**: embryonal carcinoma; Ki-67; lymph node

The 5 year survival rate for patients with metastatic non-seminomatous germ cell tumours is now over 80% with first line chemotherapy (International Germ Cell Cancer Collaboration Group, 1997). Treatment includes orchidectomy and platinum-based combination chemotherapy. Residual masses, particularly in the retroperitoneum, may remain after chemotherapy. Since the early surgical resections by Einhorn et al (1981), it has become almost routine to perform retroperitoneal lymph node dissections (RPLND) in these patients (Ravi et al, 1997). Histology from these masses has a wide spectrum of appearances including necrosis, teratoma, mature (TM), or malignant elements – embryonal carcinoma (EC) or teratoma, immature (TI). The percentage of each in one study being 45% necrosis, 42% TM and 13% malignant tumour (Steyerberg et al, 1995). These chemotherapy-treated germ cell tumours are notoriously difficult for histopathologists to analyse, when the differentiation between undifferentiated, carcinomatous, and dysplastic elements may be extremely contentious.

Few publications have considered the outcome and management of this small group of patients with viable malignant or immature tumour in RPLNDs. Because of this, the benefit of post-operative chemotherapy in these patients is controversial. The relapse rate in patients not treated with further chemotherapy from our own centre and other studies (Williams et al 1987; Tait et al 1994; Pizzocaro et al, 1998) suggests that many patients are cured by RPLND alone. It is clear that effective salvage therapy does exist for those who relapse (Loehrer et al, 1998; Shamash et al, 1999). The two alternative approaches of dealing with these patients (offer chemotherapy to everyone with residual malignant elements or only treat those who subsequently relapse) therefore overtreats or undertreats these patients.

The current policy at this centre is to offer observation initially and only offer therapy on subsequent relapse. The purpose of this study was to investigate the possibility that histological examination of the viable malignant elements and assessment of the proliferation rate could quantify the risk of relapse and thus allow for better patient selection in deciding who should have immediate treatment and for whom watchful waiting was more appropriate.

### MATERIALS AND METHODS

118 retroperitoneal lymph node dissections were identified from Barts and The London NHS Trust medical oncology database. Haematoxylin and eosin-stained slides for each case were reviewed by a consultant histopathologist, who was blinded to the outcome of the patient. Where original histopathology slides were not available, fresh sections were cut and stained and where necessary further immunochemistry performed to determine the diagnosis. The cases with malignant, immature or dysplastic elements were identified, and an assessment of the proportion of the major tissue elements was made on each case and a percentage assigned to each tissue type. This was assessed as an overall impression similar to other studies (Heidenreich et al, 1998). A paraffin block containing the malignant or immature elements was selected and used for immunohistochemical staining. 30 out of the total 118 RPLNDs had residual malignant or immature elements. Cases which had adjuvant chemotherapy before relapse were excluded so that the natural history of the disease could be determined. Cases where insufficient tissue remained or the blocks were

*Received 17 August 2000*
*Revised 23 October 2000*
*Accepted 8 November 2000*

*Correspondence to*: DM Berney

**Table 1** Retroperitoneal lymph node dissections showing different elements seen, percentage of EC, proliferation index and relapse status

| Age | Histology | % MTU | Proliferation index (%) | Relapse |
|---|---|---|---|---|
| 19 | TM, TI, EC | 1 | 0 | No |
| 33 | TM, TI, YS | 0 | 1 | No |
| 35 | TM, TI | 0 | 3 | No |
| 29 | TI (with spindle cell components) | 0 | 1 | No |
| 36 | TM, TI | 0 | 72 | No |
| 22 | EC, N | 5 | 1 | No |
| 49 | TM, TI, EC | 1 | 4 | No |
| 35 | N, TM, EC | 1 | 5 | No |
| 53 | EC | 100 | 11 | No |
| 47 | N, TI | 0 | 33 | No |
| 21 | TI | 0 | 15 | No |
| 28 | N, EC | 25 | 50 | No |
| 25 | TM, TI | 0 | 44 | No |
| 45 | TI, TM, N | 0 | 26 | No |
| 22 | N, EC | 15 | 49 | No |
| 34 | TI: neural elements and TM (glia) | 0 | 54 | No |
| 51 | EC | 100 | 55 | Yes |
| 33 | CA (clear cell carcinoma) | 100[a] | 41 | Yes |
| 28 | EC, N, TM | 5 | 75 | Yes |
| 42 | EC | 100 | 24 | Yes |
| 27 | TI, TD | 0 | 7 | Yes |
| 24 | TM, YS, EC | 10 | 33 | Yes |
| 26 | EC, TI, N | 50 | 28 | Yes |
| 21 | EC, YS, TI | 75 | 60 | Yes |
| 28 | EC, TM, N | 50 | 26 | Yes |
| 33 | EC | 100 | 59 | Yes |
| 24 | EC | 100 | 54 | Yes |
| 43 | EC, N | 90 | 16 | Yes |

[a]Carcinomatous transformation was counted as EC. EC = embryonal carcinoma; TI = teratoma, immature; TM = teratoma, mature; YC = yolk sac; N = necrosis; CA = carcinomatous transformation.

unavailable were also excluded. This left a total of 28 cases with residual malignant or undifferentiated elements (Table 1).

Formalin-fixed, paraffin-embedded 3 μm tissue sections were cut and mounted onto coated slides. Antigen retrieval by pressure cooking was used. Immunohistochemistry was performed using the ABC method for Ki-67 with the polyclonal antibody, code A047 (DAKO). A normal tonsil was used for a positive control. A negative control was performed for each case. Sections were counter-stained with Gill's haematoxylin. Ki-67 was scored as the number of positive cells out of 500 expressed as a percentage. 500 cells were counted in 10 random fields within the tumour area. Where less than 500 cells were present within a case, at least 100 cells were counted. No malignant focus included contained less than 100 cells. Patients were categorized into two groups, those that relapsed after surgery and those that remain progression free (non-relapse).

Statistical analysis was performed using JMP statistics programme, to determine significant risk factors for relapse. ShapiroWilk W test found the data unlikely to be from a normal distribution and so non-parametric Wilcoxon/Kruskal-Wallis Tests were applied.

### RESULTS

28 patients were included of whom 12 subsequently relapsed after RPLND. The mean age of the patients was 32.6. The median length of follow up of patients that remained progression free is 9.5 years, the minimum follow-up is 36 months. Of patients that relapsed, the median time after surgery to relapse is 1 month, and all others relapsed within 11 months except for 1 patient who relapsed after 30 months.

### Histology

Embryonal carcinoma (EC) was present in 11/12 of masses from relapsing patients and 7 out of 14 progression-free patients. There was a highly significant difference between the percentage of EC between the two groups ($P < 0.0006$). No difference was found between the percentage of yolk sac (YS), TM and necrosis within each group. TI showed a trend to be more common in the non-relapse group but this failed to reach significance ($P = 0.08$).

Using a cut-off point of >25% EC, 10/11 patients (90.9%) would have been correctly predicted to relapse after surgery. Using this test alone to predict the need for further chemotherapy gives a sensitivity of 75% (9/12), specificity of 93.7% (15/16) and a negative predictive value of 83.3% (15/18).

### Proliferation index

16/28 (57.1%) of cases had a Ki-67 index greater than 25%. There was a significant difference between the proliferation index in the relapse and non-relapse groups ($P < 0.03$). A cut-off value of greater than 50% would have correctly predicted 71.4% (5/7) patients that relapsed. The sensitivity was 45.5% (5/11), the specificity was 88.2% (15/17) and the negative predictive value was 71.4% (15/21).

© 2001 Cancer Research Campaign 

### Relationship between patient outcome and initial risk of relapse as assessed by the IGCCCG classification

When patient outcome was revealed the IGCCCG classification (International Germ Cell Cancer Collaborative Group, 1997) which was available for 26/28 of the patients was noted. This classification ascribes risk of relapse to the degree of elevation of tumour markers and the sites of metastatic disease. 33% (4/12) of those in the good prognostic group relapsed compared to 70% (7/10) in the intermediate group and 25% (1/4) in the poor group. Thus, there was no correlation between prognostic group at presentation and the risk of relapse in those with residual malignant elements following surgery.

### DISCUSSION

Effective salvage therapy exists for patients that relapse after a retroperitoneal lymph node dissection of a malignant residual mass (Loehrer et al, 1998; Shamash et al, 1999). Because of the rarity of metastatic germ cell tumours there are few publications correlating histology and recurrence rates in these tumours and to our knowledge, none that examine the proliferation rate in residual post-chemotherapy tumours. Immunohistochemical studies have been performed in patients with clinical stage I disease and correlated with later metastasis, but have not been investigated in the residual masses (Eid et al, 1998).

Richie and Kantoff (1991) had only 3/39 (8%) relapses in their series, while Tait et al (1984) had a relapse rate of 9/16 (56%). In the largest series (Stenning et al, 1998), 25% of all their patients (including those with teratoma, differentiated or necrosis) experienced disease progression and 60% of those with malignant elements relapsed. Persistent malignant elements and incomplete resection were independent risk factors. However 65% of patients with residual malignancy had post-resection adjuvant chemotherapy and no attempt was made to quantify the proportion of the different elements. Our series had 12/28 (42%) relapses. Patients that remain progression free, have been followed up for a minimum of 36 months.

Unsurprisingly, many papers emphasize the importance of complete resections of the masses (Fox et al, 1993; Fizazi et al, 1999; Albers et al, 2000). The malignant elements were all completely resected in our series. However, it might have been expected that patients who presented with nodal metastatic disease only (Stage 2 or 3) would be more likely to be cured by surgery than those who presented with distant non-nodal metastases. There were 20 such patients of whom 9 relapsed (8 had greater than 25% EC and 7 of these have relapsed). 8 patients had stage 4 disease at presentation and 3 relapsed. One had greater than 25% EC. Therefore the stage of the disease at presentation seems to play little part in the likelihood of relapse.

There appears to be no association between IGCCCG classification and relapse. This may be because of the small numbers of the group, but nevertheless suggests that histological parameters will assess more accurately the need for salvage chemotherapy.

The most significant finding of this study was that histopathological quantification of the proportion of residual EC is a statistically significant predictor of patient relapse ($P < 0.0006$). The presence of EC and recently the proportion of EC within orchidectomy specimens has been shown to be a significant risk factor for occult retroperitoneal disease in patients with clinical stage I disease (Heidenreich et al, 1998). In this study, 80% EC was found to have a positive predictive value of 84% for presence of metastases. Two studies, which looked at the EC component of retroperitoneal lymph nodes, produced conflicting results. Javadpour and Young (1986) found that a predominance of EC was associated with an increased relapse rate after RPLND, whereas Richie and Kantoff (1991) did not find a statistically significant association. Most of these series however are biased by the fact that chemotherapy was given to some of the patients after RPLND. This in-built bias means that these authors cannot predict the necessity for further chemotherapy after RPLND. Fizazi et al (1999) have reported a multi-centre study examining 'viable malignant cells' in RPLNDs in 140 patients, 29% of whom were not given post-surgery chemotherapy. The percentage of malignant cells was an important risk factor for relapse ($P = 0.02$). Our study is the largest single-centre series to date of RPLNDs with residual EC in which no further chemotherapy was not given until a clinically proven relapse had occurred. If our results had been applied clinically, using 25% EC as a cut-off point, we would have been able to predict 15 of the 18 patients that did not relapse and only 1 out of 10 would have received unnecessary chemotherapy. These figures alone make >25% EC appear as a good predictor of relapse. It also has the advantage of being fairly simple to measure. A higher proportion of the progression-free patients had TI present (58.8% compared to 18.2%) but this failed to achieve statistical significance ($P < 0.0855$). This may simply be a reflection of the greater EC composition in the relapse group. No association was seen between proportion of the mass that was necrotic and likelihood of relapse. Aprikian et al (1994) and Herr (1997) found necrosis seen at frozen section to be associated with a decreased tendency to relapse, allowing a limited retroperitoneal lymphadenectomy. In our experience, this is not the case, though frozen sectioning of RPLNDs is not used in our centre and the studies are therefore not easily comparable. Assessment of RPLNDs for different malignant elements is extremely difficult and this is recognized in the published literature (Stenning et al, 1998). It may be difficult to distinguish carcinomatous overgrowth from EC and also to identify extremely small foci of malignancy and to differentiate these areas from TI. This is one reason that in this study it was thought that a measurement of the proliferation index might provide more objective evidence for the likelihood of recurrence than a histopathological opinion.

The presence of 'carcinomatous' or 'sarcomatous' change within RPLNDs has been occasionally reported. Little et al (1994) found these changes in over 8% of their RPLNDs and Hines et al (1997) reported 5 sarcomas out of 88 patients, but Stenning et al (1998) none, despite searching specifically for them. In our series, there was one case of clear cell carcinoma and another case with a partially sarcomatous component which was a minority of the tumour and therefore classified as TI. The case of clear cell carcinoma was the only one of the patients that has recurred at greater than 2 years from resection and is therefore a late recurrence. Michael at al (2000) who examined a series of late recurrences of germ cell tumours, emphasized their poor chemo-responsiveness and suggested that when no teratoma was present, they be treated with surgical excision, where possible. We therefore counted this case for the purposes of analysis as EC, although they tend to be refractory to treatment with germ cell chemotherapy regimens.

Numerous reports exist on the utility of tumour proliferative activity in solid cancers for predicting prognosis (Statin et al, 1997) and most studies show Ki-67 to have greater predictive value than proliferating cell nuclear antigen (Leonardi et al, 1992).

 © 2001 Cancer Research Campaign

However, the utility of tumour proliferative activity, for predicting clinical stage I patients with metastases has produced conflicting results. Albers et al (1995, 1997) found a significant difference to exist between patients with and without metastases, whereas Heidenreich and Moul (1997) and Fernandez et al (1994) found no difference between the two groups. In this study a significantly greater ($P$ < 0.05) proliferation index was found in patients that relapsed, but the positive predictive value (PPV) was only 71.4% if a cut off of >75% was used. This compares with a PPV of 90% in Albers et al (1997) on the primary tumours. The proliferation index therefore provided significant data though appeared to be less useful than histopathological assessment.

In conclusion, proliferation rate and percentage of EC are both useful indicators for the need for further chemotherapy in patients with residual immature or undifferentiated elements in RPLNDs. The percentage of EC alone appears to be the best predictor of relapse though the proliferation index may help to identify cases with a high relapse risk, which are difficult to classify using routine techniques. These initial data may lead to a strategy to prevent the unnecessary administration of chemotherapy to patients who have undergone RPLND and whose mass contains a malignant component.

### REFERENCES

Albers P, Miller, GA, Orazi A, Ulbright TM, Albers J, Donohue JP and Foster RS (1995) Immunohistochemical assessment of tumour proliferation and volume of embryonal carcinoma identify patients with clinical stage A seminomatous testicular germ cell tumor at low risk for occult metastasis. *Cancer* **75**: 844–850

Albers P, Bierhoff E, Neu D, Fimmers R, Wernert N and Muller SC (1997) MIB-1 immunohistochemistry in clinical stage I nonseminomatous testicular germ cell tumours predicts patients at low risk for metastasis. *Cancer* **79**: 1710–1716

Albers P, Ganz A, Hannig E, Miersch WE and Muller SC (2000) Salvage surgery of chemorefractory germ cell tumors with elevated tumor markers. *J Urol* **164**: 381–384

Aprikian AG, Herr HW, Bajorin DF and Bosl GJ (1994) Resection of postchemotherapy residual masses and limited retroperitoneal lymphadenectomy in patients with metastatic testicular nonseminomatous germ cell tumors. *Cancer* **74**: 1329–1334

Eid H, Gulyas M, Geczi L, Bodrogi I, Institoris E and Bak M (1998) Expression of bcl-2 in testicular carcinoma: correlation with tumor progression and MDR1/Pgp. *Cancer* **83**: 331–336

Einhorn LH, Williams SD, Mandelbaum I and Donohue JP (1981) Surgical resection in disseminated testicular cancer following chemotherapeutic cytoreduction. *Cancer* **48**: 904–908

Fernandez EB, Sesterhenn IA, McCarthy WF, Mostofi FK and Moul JW (1994) Proliferating cell nuclear antigen expression to predict occult disease in clinical stage 1 nonseminomatous testicular germ cell tumours. *J Urol* **152**: 1133–1138

Fizazi K, Tjulandin S, Salvioni R, Ragan D, Bokemeyer C, Gerl A, De Bono JS, Flechon A, De Giorgi U, Kerbrat P, Geoffrois L, Bower M, Mahe C, Bouzy J, Bulanov A, Pizzocaro G, Hartmann JT, Nichols C, Schmoll HJ, Fossa S and Droz JP (1999) Viable malignant cells after primary chemotherapy for metastatic non-seminomatous germ cell tumours (NSGCT): results from an international study. *Proc ASCO* **18**: 308a

Fox EP, Weathers TD, Williams SD, Loehrer PJ, Ulbright TM, Donohue JP and Einhorn LH (1993) Outcome analysis for patients with persistent nonteratomatous germ cell tumor inpost chemotherapy retroperitoneal lymph node dissections. *J Clin Oncol* **11**: 1294–1299

Heidenreich A and Moul JW (1997) Immunohistochemical expression of Ki-67 to predict lymph node involvement in clinical stage I nonseminomatous germ cell tumors. *J Urol* **158**: 620–625

Heidenreich A, Sesterhenn IA, Mostofi FK and Moul JW (1998) Prognostic risk factors that identify patients with clinical stage I nonseminomatous germ cell tumours at low risk and high risk for metastasis. *Cancer* **83**: 1002–1011

Herr HW (1997) Does necrosis on frozen-section analysis of a mass after chemotherapy justify a limited retroperitoneal resection in patients with advanced testis cancer? *Br J Urol* **80**: 653–657

Hines J, Rustin G, Newlands E and Christmas TJ (1997) The prognostic significance of malignant tissue removed at retroperitoneal lymph node dissection after chemotherapy in metastatic testis cancer. *Br J Urol* **79** (Suppl 4): 21

International Germ Cell Cancer Collaborative Group (1997) International Germ Cell Consensus Classification: A prognostic factor-based staging system for metastatic germ cell cancers. *J Clin Oncol* **15**: 594–603

Javadpour N and Young JD (1986) Prognostic factors in non-seminomatous testicular cancer *J Urol* **135**: 497–499

Key G, Becker MHG, Duchrow M, Schluter C, Askaa J and Gerdes C (1991) Immunobiochemical and molecular biologic characterisation of the cell proliferation associated nuclear antigen that is defined by monoclonal antibody Ki-67. *Am J Path* **138**: 867–873

Leonardi E, Girlando S, Serio G, Mauri FA, Perrone G, Scamoni S, Dalla Palma P and Barbareschi M (1992) PCNA and Ki-67 expression in breast carcinoma: correlation with clinical and biological variables. *J Clin Path* **45**: 416–419

Little JS, Foster RS, Ulbright TM and Donohue JP (1994) Unusual neoplasms detected in testis cancer patients undergoing post-chemotherapy retroperitoneal lymphadenectomy *J Urol* **152**: 1144–1149

Loehrer PJ Sr, Gronin R, Nichols CR, Weathers T and Einhorn LH (1998) Vinblastine plus ifossfamide plus cisplatin as initial salvage therapy in recurrent germ cell tumour. *J Clin Oncol* **16**: 2500–2504

Michael H, Lucia J, Foster RS and Ulbright TM (2000) The pathology of late recurrence of testicular germ cell tumours. *Am J Surg Path* **24**: 257–273

Pizzocaro G, Nicolai N, Milani A, Piva L, Merson M, Colavita M and Salvioni R (1998) Is further chemotherapy necessary in radically resected residual cancer in non-seminomatous germ cell tumors (NSGCT) of the testis following induction chemotherapy? *Proc ASCO* **17**: 309a

Ravi R, Oliver RTD, Ong J, Badenoch DF, Fowler CG, Paris AMI and Hendry WF (1997) A single centre observational study of surgery and late malignant events after chemotherapy for germ cell cancer. *Br J Urol* **80**: 647–652

Richie JP and Kantoff PW (1991) Is adjuvant chemotherapy necessary for patients with stage B1 testicular cancer? *J Clin Oncol* **9**: 1393–1396

Shamash J, Oliver RTD, Ong J, Raja M, Edmonds P, Gallagher CJ, Ostrowski J, LeVay J and Williams M. (1999) 60% salvage rate for germ cell tumours using sequential m-BOP, surgery and ifosfamide based chemotherapy. *Ann Oncol* **10**: 685–692

Statin P, Damber J-E, Kalber GL and Bergh A (1997) Cell proliferation assessed by Ki-67 immunoreactivity on formalin fixed tissues is a predictive factor for survival in prostate cancer. *J Urol* **157**: 219–222

Stenning SP, Parkinson MC, Fisher C, Mead GM, Cook PA, Fossa SD, Horwich A, Jones WG, Newlands ES, Oliver RTD, Stenwig AE and Wilkinson P (1998) Postchemotherapy residual masses in germ cell tumor patients. *Cancer* **83**: 1409–1419

Steyerberg EW, Keizer HJ, Fossa SD, Sleijfer Dth, Toner GC, Schraffordt Koops H, Mulders PFA, Messemer JE, Ney K, Donohue JP, Bajorin D, Stoter G, Bosl GJ and Habbema JDF (1995) Prediction of residual retroperitoneal mass histology after chemotherapy for metastatic nonseminomatous germ cell tumor: multivariate analysis of individual patient data form six study groups. *J Clin Oncol* **13**: 1177–1187

Tait D, Peckham MJ, Hendry WF and Goldstraw P (1984) Post-chemotherapy surgery in advanced non-seminomatous germ cell testicular tumours: the significance of histology with particular reference to differentiated (mature) teratoma. *Br J Cancer* **50**: 601–609

Toner GC, Panicek DM, Heelan RT, Gellere NL, Lin S-Y, Bajorin D, Motzer RJ, Scher HI, Herr HW, Morse MJ, Fair WR, Sogani PC, Whitmore WF, McCormack PM, Bains MS, Martini N and Bosl GJ (1990) Adjunctive surgery after chemotherapy for nonseminomatous germ cell tumors: recommendations for patient selection. *J Clin Oncol* **8**: 1683–1694

Williams SD, Stablein DM, Einhorn LH, Muggia FM, Weiss RB, Donohue JP, Paulson DF, Brunner KW, Jacobs EM, Spaulding JT, DeWys WD and Crawford ED (1997) Immediate adjuvant chemotherapy versus observation with treatment at relapse in pathological stage II testicular cancer. *N Engl J Med* **317**: 1433–1438

*© 2001 Cancer Research Campaign*
